# Addressing Emotional and Behavioral Symptoms in Young Children: The Potential of Non-Therapeutic Play and Art

**DOI:** 10.3390/children12050551

**Published:** 2025-04-25

**Authors:** Alexander Veraksa, Valeriya Plotnikova, Dmitry Kornienko, Natalia Rudnova, Margarita Gavrilova

**Affiliations:** Laboratory of Childhood Psychology and Digital Socialization, Federal Scientific Center of Psychological and Multidisciplinary Research, Moscow 125009, Russia; veraksa@yandex.ru (A.V.); corney@yandex.ru (D.K.); rudnova.na@yandex.ru (N.R.); gavrilovamrg@gmail.com (M.G.)

**Keywords:** pretend play, project approach, anxiety, withdrawal, aggressive behavior, emotional symptoms, behavioral symptoms, early childhood

## Abstract

**Background/Objectives**: The search for non-therapeutic ways to reduce emotional and behavioral symptoms is an important task. The aim of this study was to assess the potential of pretend play and art-based project activity as easily implementable ways to reduce anxiety, aggression, and anti-social behavior in preschoolers. **Methods**: A total of 36 preschoolers (mean age 68.7 months) with high anxiety–withdrawal level were selected and divided into four groups: adult-supported pretend play, free pretend play, project activity, and a control group. Each group had 20 sessions, lasting 20–25 min. Pre- and post-test included the assessment of anxiety–withdrawal, anger–aggression, and social competence. Executive functions were also assessed at the pre-test as a control variable. **Results**: The results showed that art-based project activities reduced anxiety–withdrawal in preschoolers. Pretend play, both with and without adult involvement, did not have a significant effect. No significant changes were found for anger–aggression and social competence. Results revealed that the level of executive functions was a significant predictor of the reduction in anxiety–withdrawal. **Conclusions**: The study specified the role of executive functions in emotional symptoms and showed the potential of art-based project activities to reduce anxiety. The results obtained can be implemented in kindergarten practice.

## 1. Introduction

The preschool age is the most intensive period for emotional and behavioral development [1,2,3]. On the one hand, emotional and behavioral development is highly sensitive to external and internal factors in children 3–7 years old [1,4]. On the other hand, emotional development in this period appears to be a protective resource and crucial predictor of future well-being and resilience [5,6].

Nowadays, children are facing rising pressure from normative (which happen to the majority of children, such as transition from the kindergarten to school, new educational clubs, etc.) and nonnormative stressful events (which do not happen to the majority of children, such as parent divorce, forced relocation, chronic illness, etc.), which threatens their emotional and behavioral development. Moreover, anxiety ranks as the second most prevalent mental health disorder among children, affecting up to 9% of preschoolers worldwide [7,8]. There is an increasing number of children who exhibit emotional and behavioral symptoms in preschool age such as anxiety, withdrawal, aggressive behavior, anti-social behavior, and others, which in adulthood can lead to emotional and behavioral problems, disorders, and a decrease in overall well-being [9]. Numerous studies have examined the consistency of psychological symptoms in children. For example, Edwards et al. [10] reported a notably high level of stability in anxiety symptoms (r = 0.75) among children aged 3 to 5 over a year. Other studies indicate that externalizing problems also tend to remain stable throughout both preschool and school years [11,12,13]. While some preschool children may eventually overcome transient emotional and behavior symptoms, there is a significant number of children who experience difficulties that serve as predictors of long-term maladjustment.

The preschool years are considered a critical period for identifying and addressing early signs of emotional and behavioral difficulties before they evolve into chronic issues. Studies highlight the essential role of the early childhood education system in establishing effective prevention and early intervention approaches [14]. Unfortunately, many children experiencing severe emotional and behavioral symptoms do not receive professional support [15,16,17,18]. Families may not perceive the symptoms as concerning until the symptoms create strain for parents, teachers, or others [17,18]. Therefore, parents and teachers rarely consider internalizing symptoms such as anxiety as requiring psychological intervention. To sum up, there is a particular interest in identifying corrective and preventive methods for addressing negative emotional and behavioral symptoms, in particular anxiety, and promoting emotional development in preschool-aged children that are applicable in large-scale early childhood institutions.

### 1.1. Play as a Tool for Overcoming Emotional and Behavioral Symptoms

In modern clinical and psychotherapeutic practice, play therapy is commonly implemented to alleviate emotional and behavioral difficulties in children [19,20]. Play therapy is defined as a “dynamic system of interpersonal relationships between a child and a therapist trained in play therapy procedures (e.g., therapeutic play), who provides the child with symbolic play materials and facilitates the building of a safe relationship so that the child can fully express and explore their own self: feelings, thoughts, experiences, and behaviors, through play—the child’s natural means of communication” [21]. The key mechanisms in play therapy include communication, particularly the expression and processing of emotions and thoughts related to the stressful event through symbolic means, as well as enhancing a sense of control, autonomy, and success [22,23,24,25]. However, organizing play therapy requires deep specialist training and long-term sessions, which limits their accessibility in public children’s institutions [23,24,25].

At the same time, similar mechanisms that contribute to reduced emotional and behavioral symptoms in play therapy can be involved in play outside of the therapeutic setting, particularly in pretend play. Pretend play is a culturally conditioned, leading form of activity for preschool children, in which they reproduce various areas of real life and the “adult world” in imaginary situations through plots and roles, mastering social relationships and interaction skills [26,27]. In pretend play as well as in play therapy, children express their personal meanings and experiences (*perezhivanie*) [28] with symbolic means [29], which helps to work through negative emotions. Imaginary situations provide children opportunities to experiment with their social behavior and emotional responses, allowing children to explore different options for emotional and behavioral strategies [29]. Assuming a role involves following rules, which promotes the development of self-regulation, including emotional self-regulation [30]. Research claims that pretend play is an effective way to develop children’s emotion self-regulation and control of destructive anti-social behavior [31,32,33]. In pretend play, the child demonstrates internal activity and a degree of independence by determining the development of the plot and their role, which contributes to the child’s sense of control and autonomy [34,35]. Thus, pretend play has therapeutic potential to reduce emotional and behavioral symptoms in children. The spontaneous character of pretend play in children and the opportunity to use substitute objects in pretend play make it accessible and easy to organize in various conditions. However, this hypothesis requires further validation.

### 1.2. Art as a Tool for Overcoming Emotional and Behavioral Symptoms

Art therapy is also considered a way of overcoming negative emotional and behavioral symptoms and coping with stress [36,37]. Art therapy utilizes the expressive nature of creating visual art within a therapeutic relationship to support and facilitate individual psychological processes [38]. Interaction with art and creativity allows one to reflect emotions and experiences on a symbolic level and transform them. Various forms of art therapy exist and may include drawing, coloring, photography, sculpture, dance, creative writing, and storytelling, but they are all rooted in creativity [39]. The key pathways for reducing emotional and behavioral symptoms in art therapy include the controlled symbolic expression and regulation of emotions, the sense of personal freedom and success during creative activities, the integration of experiences into a broader autobiographical narrative, and the opportunity to acquire new skills [40]. Despite the relative accessibility of some individual art therapy techniques, comprehensive programs require extensive and long-term specialist training. In most European countries, art therapists complete a minimum of two years of postgraduate education to become certified as state-registered healthcare professionals. This imposes limitations on the use of art therapy in public kindergartens.

However, similar mechanisms can be found in other creative forms of working with art, for example, in art-based project activities [41]. Art-based project activity primarily focuses on the creation of a new product (e.g., crafts, books, performances, illustrations, video) that reflects the child’s personal vision. However, its execution unfolds as a problem-solving situation where direct, straightforward action is impossible [42,43]. This prompts the child to seek ways to overcome obstacles and achieve greater emotional relief. Art-based project activity is a productive form of supportive interaction between the adult and the child [41,44]. Within the framework of the project, the adult encourages the child’s initiative. The child demonstrates creativity and initiative, and with the support of the adult, shapes it into a socially acceptable product. The child receives an optimal balance of freedom and guidance from the adult [45]. In this way, personal and social meanings, experiences, and ideas of the child are realized, often through the use of symbolic means such as drawing, performing a play, making crafts, etc. Art-based project activity allows the child to expand their experience and social interaction skills, express personal ideas and feelings, and gain control and reframe previous emotional experiences. Moreover, art-based project activity is easy to organize within a group setting and does not require special training for educators, as they act as helpers and interpreters for the children [46]. Thus, art-based project activity incorporates many of the mechanisms found in art therapy. Additionally, a project can be organized by regular preschool educators within a group setting. This suggests that art-based project activity may help alleviate negative emotional and behavioral symptoms such as anxiety and aggression in children. However, this assumption also requires further empirical validation.

### 1.3. Research Problem

The main objective of this research is to identify easily implementable non-therapeutic ways to reduce negative emotional and behavioral symptoms in preschoolers, specifically within the group setting of a kindergarten. Anxiety, aggression, and anti-social behavior—being the most common emotional and behavioral symptoms—are in particular focus [9,47]. The tools explored in this study are pretend play and art-based project activity, which are similar in their mechanisms of influence on the mental state to play therapy and art therapy but are organized outside the therapeutic context. Therefore, the first objective of this research is to assess the potential for applying pretend play and art-based project activity to common emotional and behavioral symptoms in preschoolers. The second objective is to analyze the comparative effectiveness of these two types of activities.

The following hypotheses have been put forward:

1. Pretend play and project activity will reduce emotional and behavioral symptoms in young children. This assumption was put forward because these non-therapeutic activities are similar in their mechanisms of influence on psychological processes to play and art therapy, respectively.
2. Pretend play with an adult will be more effective in reducing anxiety and withdrawal, anger and aggressive behavior, and developing children’s social competence than playing without an adult. The assumption is based on the idea of the adult role for the child’s mental development in play as a carrier of cultural means and forms [27,48].
3. Pretend play with an adult will be more effective in reducing anxiety and withdrawal, anger and aggressive behavior, and developing children’s social competence than project activity because play is the leading and most developmental activity in preschool age [26].

## 2. Materials and Methods

### 2.1. Participants and Procedures

To achieve the research goals and objectives, a randomized controlled experiment with pre- and post-test was conducted, consisting of four stages.

In the first stage, 134 preschoolers aged 5–6 years (mean age 69.5 months) were assessed for anxiety–withdrawal, anger–aggression, and social competence. Additionally, executive functions (EF) were evaluated as a control variable. The assessment was conducted on an individual basis with each child. After the EF assessment, the children were divided into levels according to the results of the cluster analysis (K-means clustering): low and high EF levels. All participants attended public kindergartens in Moscow. The areas where the kindergartens were located are characterized by similar infrastructure levels and are designed for families with a middle income level. The parents of all children participating in the study provided written consent. The study was approved by the Ethics Committee of the Federal Scientific Center of Psychological and Multidisciplinary Research (Ethic approval No. 2, 31.01.2024).

In the second stage, 25% (upper quartile) of children with the highest scores for anxiety–withdrawal were selected. Anxiety—withdrawal was chosen as the selection criterion since the average scores and maximum for this indicator were higher than for anger–aggression. All children whose scores matched the lower boundary of the upper quartile were also included in the experimental part of the study. Thus, 36 preschoolers with the most pronounced symptoms of anxiety–withdrawal were selected and randomly assigned to four groups: adult-supported pretend play (N = 9), free pretend play (N = 9), art-based project activity (N = 9), and a control group (N = 9). The groups were equalized by gender and the number of children with the same EF level.

In the third stage, each experimental group participated in 20 sessions, each lasting 20–25 min. Sessions were held twice a week in additional activity rooms at the kindergartens. The sessions were conducted by specially trained psychologists and were completed simultaneously across all groups. No special activities were organized for the control group.

In the fourth stage, a post-test assessment of anxiety–withdrawal, anger–aggression, and social competence, similar to the initial diagnostics, was conducted. The final research sample included 29 preschoolers (mean age 68.7 months, 65.5% boys), who attended more than half of the sessions and participated in the post-test: adult-supported pretend play (N = 6), free pretend play (N = 8), art-based project activity (N = 9), and a control group (N = 6). Despite that the number of participants in each compared group in the final sample is small—from 6 to 9—it is assumed that a group of 6 participants is enough for stimulating cognitive and emotional internal processes (e.g., creativity, memory, emotional enthusiasm) [49,50].

The groups in the final sample did not differ in the number of children with low and high levels of EF (χ^2^(3) = 1.65, *p* = 0.648) and gender composition (χ^2^(3) = 0.967, *p* = 0.809). Data on the gender and EF composition of the final sample are presented in Appendix A (Table A1).

### 2.2. Measures

The Russian adaptation of the “Social Competence and Behavior Evaluation (SCBE-30)” questionnaire for educators was used to asses anxiety–withdrawal, anger–aggression, and social competence [51]. Each scale includes 10 items that describe the emotional and behavioral symptoms in children. Educators assess the frequency of manifestation of each characteristic in a child on a scale from 1 to 6, as follows: 1—such behavior is never observed; 2–3—sometimes occurs; 4–5 is common; 6—is always observed. Cronbach’s coefficient alpha for anxiety–withdrawal 0.82, anger–aggression 0.83, and social competence 0.79.

According to A. Miyake’s EF model [52], three components of the EF were evaluated: working memory, inhibitory control, and cognitive flexibility, using the NEPSY–II subtests [53] adapted for the Russian-speaking sample [54,55,56]. Namely, the assessment of visual working memory was carried out using the “Memory for Designs” test (reliability, retest correlation, r = 0.76), in which the child needs to memorize and reproduce pictures and locations in a limited time and in the presence of distractors. The “Sentence repetition” test (reliability, retest correlation, r = 0.77) was used to evaluate auditory working memory. The child is required to repeat sentences, gradually becoming more complex grammatically and lexically, after an adult. To measure cognitive inhibitory control, we used the “Naming and Inhibition” test (reliability, retest correlation, r = 0.81 for naming, r = 0.79 for inhibition). Initially, the child must correctly name figures presented on a field, with the completion time reflecting their information processing speed. Subsequently, in the reverse task, if squares and circles are depicted, the child must call squares “circles” and circles “squares” in the second part. This section assesses the ability to suppress impulsive reactions. Additionally, the “Dimensional Change Card Sort” test (reliability, retest correlation, r = 0.71) was applied to evaluate cognitive flexibility, requiring the child to sort cards according to changing rules [56].

### 2.3. Study Groups

The study involved four groups: three experimental groups—adult-supported pretend play, free pretend play, project activity—and one control group. To evaluate the role of the adult in pretend play for reducing emotional and behavioral symptoms in children, two play groups were organized: one with full adult participation as a player, and the second with minimal adult involvement in the play.

In the adult-supported pretend play group, children read the stories “Pinocchio” (1–10 play sessions) and “The Wizard of the Emerald City” (11–20 play sessions) in parts, which served as a starting point for the pretend play. However, preschoolers were not required to strictly follow the plot of the stories. These stories were selected because their narrative can be related to real-life situations where a child faces high uncertainty, negative emotions (sadness, anger, etc.), friendship, and family support: the hero is separated from their family, encounters challenges, makes new friends, receives help, and overcomes trials. Additionally, the play space was filled with open-ended play materials.

In the free pretend play group, the experimenters also enriched the play space with open-ended, non-play materials such as sticks, cones, boxes, leaves, and others. They did not participate in the children’s activities and refrained from intervening in the play. However, they could assist the preschoolers in initiating the play by introducing them to the environment, facilitating discussions on the theme of the play and roles, answering children’s questions, and ensuring the safety of the environment.

The art-based project activity group focused on the creation of a new product. They staged a theatrical performance based on a script written by the children themselves. The project result (the theatrical performance) was selected through a discussion of ideas proposed by the group members, organized within a problem-solving context. The children suggested their ideas, sketches for the decorations, and a draft plan for the performance script, after which one of the proposed options was chosen by voting. Most of the classes were devoted to creating a performance plot. The adult supported and revealed the initiatives of the children, discussed the emotions of the characters in the performance, directed the plot to the topics of overcoming difficulties, experiencing realistic emotions, and helping. The children used visual materials to create decorations in a symbolic way, such as magic pollen, giving strength, or amulets of kindness. Once the project’s goal was determined, the participants moved on to execution: technical construction of the crafts and decorations, writing the play, preparing props, learning lines, and rehearsing. Upon completion, the group presented their projects at the kindergarten.

## 3. Results

### 3.1. Descriptive Statistics

For the final sample, no significant differences were found between the groups for anxiety–withdrawal and anger–aggression scales. (Kruskal–Wallis, *p* > 0.05). However, in the adult-supported pretend play group, social competence was significantly higher compared to the other groups. (Kruskal–Wallis, χ^2^(3) = 10.24, *p* = 0.017). Descriptive statistics for the final sample at pre- and post-test are presented in Appendix A (Table A1).

### 3.2. Effectiveness Analysis

To evaluate the effectiveness of each type of intervention and the change from pre-test to post-test in groups, the Wilcoxon test was conducted. A significant reduction for anxiety–withdrawal scores was revealed in the art-based project activity group (W = 44, *p* = 0.013, r = 0.956). For other conditions and parameters, no significant changes were found.

Given the differences between the groups at the pre-test stage, the comparison of the conditions was performed by analyzing differential scores, i.e., the change in each indicator’s scores from pre-test to post-test, using the Kruskal–Wallis test. The results showed that the groups significantly differed in the change of anxiety–withdrawal scores (Kruskal–Wallis test, χ^2^(3) = 10.95, *p* = 0.012, ε^2^ = 0.39). Pairwise comparisons using the Dwass–Steel–Critchlow–Fligner (DSCF) test identified differences between the art-based project activity group and the adult-supported pretend play group at a trend level (DSCF, W = 3.6, *p* = 0.053), as well as between the art-based project activity group and the free pretend play group (DSCF, W = 3.8, *p* = 0.035). In both cases, the reduction in anxiety–withdrawal in the art-based project activity group was more significant than in the play groups. No significant differences were found for the anger–aggression or social competence scores.

To clarify the factors influencing the reduction of anxiety–withdrawal in preschoolers, a regression analysis was conducted. The dependent variable was the differential change in participants’ anxiety–withdrawal scores. The factors included were the level of EF (reference level: low EF) and study group (reference level: control group). The best-fitting model explained 94% of the variance in the dependent variable (R^2^ = 0.943, F(8, 10) = 20.6, *p* < 0.001), as shown in Table 1.

**Figure 1:**
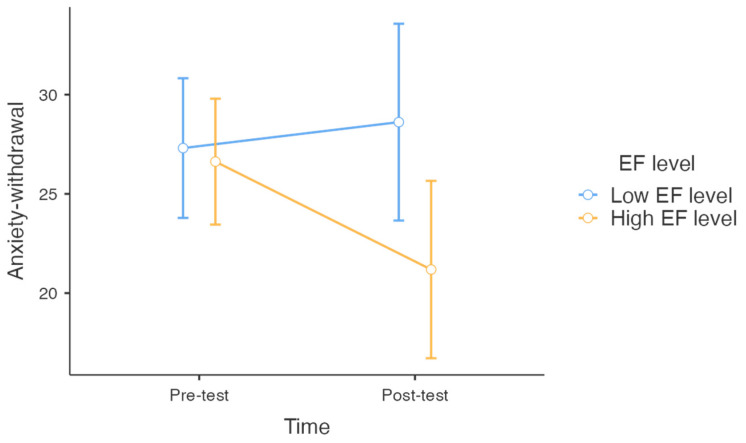
Mean anxiety–withdrawal scores at pre-test and post-test for children with different levels of executive functions (EF).

The regression analysis indicated that changes in anxiety–withdrawal scores were influenced by the study group (Omnibus test, ANOVA, F(3) = 6.96, *p* = 0.008) and the level of EF in preschoolers (Omnibus test, ANOVA, F(1) = 10.06, *p* = 0.01). Specifically, participation in the art-based project activity group significantly contributed to a reduction in anxiety–withdrawal compared to the control group (t = −2.86, *p* = 0.017, β = −0.83), while other conditions did not prove significant differences compared to the control group (t = −1.7, *p* = 0.119, β = −0.5 for adult-supported pretend play; t = 0.177, *p* = 0.853, β = 0.56 for free pretend play). Preschoolers with high EF levels showed a greater reduction in anxiety–withdrawal than those with low EF levels (t = −3.172, *p* = 0.01, β = −0.6, see Figure 1). The analysis also revealed that post-test anxiety–withdrawal scores (t = 6.455, *p* < 0.001, β = 0.6), post-test social competence scores (t = 2.488, *p* = 0.032, β = 0.25), post-test information processing speed (t = 4.14, *p* = 0.002, β = 0.38), and pre-test verbal-auditory working memory (t = −4.11, *p* = 0.002, β = −0.43) were significant predictors of changes in anxiety–withdrawal. Other factors, including pre-test anxiety–withdrawal levels, were not identified as significant predictors.

## 4. Discussion

The main aim of this study was to evaluate and compare the effectiveness of two non-therapeutic approaches—pretend play and art-based project activity—in alleviating emotional and behavioral symptoms in preschool children. The results of the experiment showed that art-based project activity had a significant impact on reducing anxiety–withdrawal in preschoolers, which is consistent with other studies on the use of art and creative activities in therapy to reduce anxiety in children [39,57]. The obtained data suggest that art-based project activity can be effectively implemented in preschool settings as a viable alternative to art therapy. In contrast, pretend play, both with and without adult involvement, did not show a significant effect. What factors could have influenced the results obtained in the experiment?

Art-based project activity, like pretend play, offers opportunities for safe emotional expression, validation, and overcoming negative emotions through symbolic means [40]. At the same time, art-based project activity provides a much greater opportunity for the use of visual art materials for emotional expression and transformation of emotions and experiences. Art materials can be easily and visibly altered. The tangible transformation of real objects is an essential step in mastering internal processes and actions in children [58,59]. The transformation of emotional experiences in a material way contributes to their reduction and the development of cultural means of emotional regulation in art-based project activities. In art-based project activity, the adult facilitates reflection on personal meaning and relationships [42,43], which contributes to a better awareness of the child’s inner feelings, whereas in pretend play adult actions are not aimed at understanding personal meaning. In pretend play, the adult is focused on developing the game plot and helps to keep children in their roles.

Another key distinction between project activity and pretend play is the degree of child agency and the focus of the activity. First, art-based project activities are designed to foster children’s initiative and support their ideas [41,44]. In project activity, the child has the highest level of authorship, experiencing their ideas as socially meaningful, important, and interesting, which may contribute to reducing anxiety. In contrast, in pretend play, the child symbolically reenacts real-world adult situations [26,43]. In pretend play, the child must understand the cultural meanings embedded in these situations. As D.B. Elkonin wrote, “…the content of a fully developed pretend ply is not the object and its use or transformation by humans, but rather the relationships between people, realized through actions with objects; not human-object, but human-human” ([26], p. 31). Therefore, even in free pretend play, the child remains constrained by cultural meanings, which limits their autonomy and sense of agency.

Secondly, in project activity, the child is result-oriented. Project activity fosters creative initiative, and as L.S. Vygotsky wrote, “Creative imagination, in its complete form, seeks external confirmation through a result that exists not only for the creator but also for others” ([60], pp. 36–37). In other words, a child’s creative idea requires realization in a tangible product and recognition of its significance by other people, which can be achieved through project activity. A focus on results carries emotional risks; for example, the child may fear disapproval or making mistakes. However, supporting initiative, providing emotional encouragement during the creation process, eliminating the concept of “right” and “wrong” ideas, and fostering social acceptance through positive interactions with adults and peers in project activity can mitigate these emotional risks and offer a positive experience. Together, these factors can help reduce a child’s anxiety related to social expression and fear of making mistakes. At the same time, pretend play is a non-productive activity. It emerges because a child, due to their developmental stage, is unable to fully participate in “adult” types of activities, despite having a need to be involved in such activities [26,43]. For instance, a study by Lillard and Taggart demonstrated that children preferred engaging in real “adult” activities compared to play because they enjoyed feeling effective and useful [61]. As a result, one of the defining characteristics of play is its process-oriented nature. While pretend play provides opportunities for positive social interactions, it does not offer recognition of the child’s activity as socially meaningful due to its non-productive nature. Thus, the productive nature of project activity, the high degree of child agency in it, and the supportive role of adults expand children’s opportunities for group inclusion, which can help reduce withdrawal in the kindergarten setting.

To sum up, art-based project activity allows children to express and reframe previous emotional experiences through visual art materials, express personal ideas and feelings, develop a sense of agency and self-worth, gain social recognition, and engage in activities typically associated with the “adult world”. Together, these factors may explain the greater effectiveness of art-based project activities compared to pretend play in reducing anxiety and withdrawal.

Despite the positive effect of art-based project activity on reducing anxiety and withdrawal, none of the proposed approaches contributed to lowering levels of anger–aggression or improving social competence. Previous studies suggest that project-based activities develop social skills [46,62,63,64]. For example, in the study by Farida N. and Rasyid H. [62], it was shown that after a course of project-based activities, preschool children aged 5–6 demonstrated increased tolerance, social politeness, and respect for others. Similarly, the study by Subagyo [64] highlighted positive effects of project activity on children’s ability to respect each other, take responsibility for others, and show empathy. As demonstrated in previous studies, project-based activities contribute to creating a more positive group climate and fostering intergroup contact, increasing the levels of acceptance, respect, and empathy among children. Notably, in the research by Sportelli et al. [65], it was shown that digital intergroup contact and empathy for hate speech victims may foster counter-speech responses and contribute to creating a safer group environment. This effect was achieved through the introduction of digital agents who helped users to collaboratively overcome challenges and encouraged awareness of hate speech’s negative consequences while promoting counter-speech strategies. In project-based activities, this role is fulfilled by an adult, who facilitates joint discussions, collaborative work, and positive communication among children. Drawing parallels with the aforementioned studies, it can be assumed that project activity helps children develop empathy, respect, and tolerance, thereby fostering more positive group interactions. This, in turn, helps children feel more confident and secure. This could explain why, in the current work, children who engaged in art-based project activity did not increase social competence but still experienced reduced anxiety and withdrawal. Meanwhile, for overcoming aggression, training aimed at developing specific social skills and anger control may be more promising [66,67]. At the same time, the obtained result may be due to the study design and the selection of only children with high anxiety–withdrawal, as well as the division of preschoolers into study groups based on this particular indicator.

This study did not find a positive effect of pretend play in overcoming emotional and behavioral symptoms in children with high anxiety. However, research on play therapy suggests that it can reduce anxiety, negative emotions, and aggression, as well as improve social skills and academic performance in children with anxiety disorders or high personal anxiety [22,23,24,25]. At the same time, a substantial body of research demonstrates the effectiveness of various types of play—such as pretend play, play with rules, and digital play—in providing temporary relief from anxiety in stressful situations, such as blood draws, adaptation to a new kindergarten, or dental procedures [68]. Studies indicate that the theme and materials of the play should be closely aligned with the underlying causes of negative emotional symptoms and should include tasks that promote emotional understanding [68]. In the present study, although storybook themes were selected to reflect common childhood emotional challenges and experiences, this was insufficient to achieve a therapeutic effect in adult-supported pretend play. It appears that for pretend play to be effective in addressing negative emotional and behavioral symptoms, its plot must be more specifically tailored to the target emotional or behavioral symptom.

Finally, it was shown that the level of EF is a significant predictor of the reduction in anxiety–withdrawal: children with higher self-regulation exhibited a greater decrease in this indicator. Specifically, information processing speed and verbal-auditory working memory played a key role in this process. EF are closely linked to emotional regulation and develop in tandem [69]. Previous studies have demonstrated that cognitive inhibition is required for emotional regulation between the ages of 4 and 6 [52,69]. It has also been found that participants with higher EF levels show a better ability to regulate emotions compared to those with lower EF levels [70]. Neuroscientific data have shown that the brain regions responsible for working memory are involved in tasks related to emotional perception and understanding [71,72]. Both attention control and emotion control share key requirements: preventing impulsive reactions and executing the opposite action. It appears that a high level of EF is associated with the ability to regulate emotional responses, including anxiety. Therefore, preschoolers with higher EF levels demonstrated a greater reduction in anxiety–withdrawal in this study. These findings are consistent with previous research and also extend it by highlighting the quality of the relationship between EF and anxiety in preschool-aged children.

A key limitation of this study is the relatively small sample size, which prevents the authors from drawing broad conclusions. Nevertheless, complementary statistical methods were employed to provide the most reliable conclusions in this case. Future studies should expand the sample and clarify the demographic variables (e.g., socioeconomic status, cultural background) as well as explore how different non-therapeutic activities, including digital apps, influence socio-emotional indicators (empathy, subjective well-being, confidence, etc.) in children with emotional and behavioral symptoms. Another important limitation is the division of children into groups based on one indicator: anxiety–withdrawal, while other emotional and behavioral symptoms were not taken into account. This design makes it difficult to draw conclusions about various emotional and behavioral symptoms, but it reflects the specifics of working with anxiety and detachment.

This study compared the effectiveness of adult-supported pretend play, free pretend play, and art-based project activity in overcoming emotional and behavioral symptoms in preschoolers. It was shown that art-based project activity contributes to reducing anxiety and withdrawal. However, none of the interventions reduced aggression or social difficulties. The findings provide a basis for future research endeavors, specifically, to find non-therapeutic approaches to reduce aggressive behavior in children, to implement art-based project activities for children with high behavioral symptoms and/or aggressive behavior, and to analyze the specifics of play as a leading activity in preschool age to overcome behavioral and emotional symptoms. It was also found that a high level of EF in children contributes to a reduction in anxiety. This highlights the preventive potential of activities promoting EF to reduce emotional and behavioral symptoms. It is necessary to study in more detail the impact of training aimed at developing self-regulation, in particular EF, on reducing anxiety and other emotional and behavioral symptoms in children. To conclude, the study showed that art-based project activities have the potential to reduce anxiety and withdrawal in young children and can be implemented in kindergarten practice as a convenient and accessible approach. Developing EF can facilitate the process of reducing anxiety. Nevertheless, developing methods to reduce emotional and behavioral symptoms that are easy to organize in kindergarten groups remains an important task.

## Figures and Tables

**Table 1:** Results for the model predicting anxiety–withdrawal gains after training.

Regression Best Fit Model	Weight	SE	t	*p*	β
Constant:	−9.111	5.6767	−1.605	0.140	
Condition:					
Art-based project activity group—control group	−6.493	2.2684	−2.862	0.017	−0.8341
Adult-supported pretend play group—control group	−4.622	2.7131	−1.704	0.119	−0.5938
Free pretend play group—control group	0.441	2.4922	0.177	0.863	0.0566
EF Level					
High EF level— Low EF level	−4.706	1.4835	−3.172	0.010	−0.6045
Predictors					
Anxiety–withdrawal, post-test	0.497	0.0770	6.455	<0.001	0.6088
Social competence, post-test	0.263	0.1056	2.488	0.032	0.2526
Verbal-auditory working memory, pre-test	−0.973	0.2368	−4.110	0.002	−0.4335
Naming, post-test	0.924	0.2230	4.142	0.002	0.3860

## Data Availability

The full dataset is available upon request to the corresponding author.

## Abbreviations

The following abbreviations are used in this manuscript:

DSCF	Dwass–Steel–Critchlow–Fligner test
EF	Executive functions

## Appendix A

**Table A1:** Descriptive statistics for assessed parameters at pre- and post-test for experimental and control groups.

		Pre-Test			Post-Test		
Parameters	Study Group	Median	Standard Deviation	χ^2^ Kruskal–Wallis, p	Median	Standard Deviation	χ^2^ Kruskal–Wallis, p
Anxiety–withdrawal	Adult-supported pretend play t (N = 6, 50% boys)	24.5	8.27	χ^2^(3) = 4.56, *p* = 0.207	27.5	11.3	χ^2^(3) = 6.72, *p* = 0.081
	Free pretend play (N = 8, 75% boys)	25.5	4.81		25.5	7.79	
	Project activity (N = 9, 66% boys)	28	6.39		19	4.36	
	Control group (N = 6, 66% boys)	22.5	3.94		21	11.9	
Anger–agression	Adult-supported pretend play t (N = 6, 50% boys)	17.5	7.94	χ^2^(3) = 1.97, *p* = 0.578	15.5	2.48	χ^2^(3) = 6.17, *p* = 0.103
	Free pretend play (N = 8, 75% boys)	17.5	9.75		17.5	7.95	
	Project activity (N = 9, 66% boys)	24	5.75		19	7.7	
	Control group (N = 6, 66% boys)	16.5	6.99		23.5	4.94	
Social competence	Adult-supported pretend play t (N = 6, 50% boys)	50	7.87	χ^2^(3) = 10.24, *p* = 0.017	43.5	5.96	χ^2^(3) = 11.33, *p* = 0.01
	Free pretend play (N = 8, 75% boys)	28	11.9		32	7.69	
	Project activity (N = 9, 66% boys)	31	7.58		33	4.72	
	Control group (N = 6, 66% boys)	27	7.57		28.5	8.18	
